# Relating Language and Music Skills in Young Children: A First Approach to Systemize and Compare Distinct Competencies on Different Levels

**DOI:** 10.3389/fpsyg.2016.01616

**Published:** 2016-10-25

**Authors:** Caroline Cohrdes, Lorenz Grolig, Sascha Schroeder

**Affiliations:** Max Planck Research Group ‘Reading Education and Development’, Max Planck Institute for Human DevelopmentBerlin, Germany

**Keywords:** language, music, children, skill acquisition, cognitive processing

## Abstract

Children in transition from kindergarten to school develop fundamental skills important for the acquisition of reading and writing. Previous research pointed toward substantial correlations between specific language- and music-related competencies as well as positive transfer effects from music on pre-literacy skills. However, until now the relationship between diverse music and language competencies remains unclear. In the present study, we used a comprehensive approach to clarify the relationships between a broad variety of language and music skills on different levels, not only between but also within domains. In order to do so, we selected representative language- and music-related competencies and systematically compared the performance of *N* = 44 5- to 7-year-old children with a control group of *N* = 20 young adults aged from 20 to 30. Competencies were organized in distinct levels according to varying units of vowels/sounds, words or syllables/short melodic or rhythmic phrases, syntax/harmony and context of a whole story/song to test for their interrelatedness within each domain. Following this, we conducted systematic correlation analyses between the competencies of both domains. Overall, selected competencies appeared to be appropriate for the measurement of language and music skills in young children with reference to comprehension, difficulty and a developmental perspective. In line with a hierarchical model of skill acquisition, performance on lower levels was predictive for the performance on higher levels within domains. Moreover, correlations between domains were stronger for competencies reflecting a similar level of cognitive processing, as expected. In conclusion, a systematic comparison of various competencies on distinct levels according to varying units turned out to be appropriate regarding comparability and interrelatedness. Results are discussed with regard to similarities and differences in the development of language and music skills as well as in terms of implications for further research on transfer effects from music on language.

## Introduction

Language and music skills seem to be linked on different levels and according to different research perspectives. This position is supported by studies investigating interrelationships between the two domains as well as from research focusing on transfer effects from one domain to another. In particular, young children in preschool seem to benefit from training musical skills which show positive transfer effects on pre-literacy skills and literacy acquisition in school (cf. meta-analysis by Butzlaff, 2000). In this paper, we want to contribute to this line of research by providing a systematic description of the relationships between various music and language skills and by identifying potential key competencies for the further promotion of language and music skill acquisition. To do this, we first briefly discuss previous findings on the interrelationships between language and music skills and skill acquisition. After that we describe the rationale of the present study and introduce our hypotheses.

### Interrelation of Language and Music Skills

Several studies have examined potential relationships between music and language skills and have shown that there are significant positive correlations for various competencies, such as e.g., pitch and phonemic discrimination (PD; Lamb and Gregory, 1993; Anvari et al., 2002), rhythm and prosody (e.g., Patel et al., 2006), rhythmic abilities and reading (e.g., Douglas and Willats, 1994) and the recall of melodies and sentences (e.g., Harms et al., 2014). Most of these studies, however, are correlational but there are also experimental studies that investigated the impact of musical training on language skills. For example, Strait et al. (2011) showed that musical aptitude was a significant predictor and accounted for over 40% of the variance in reading performances in children from 8 to 13 years with little to no music training. In addition, they could show that the relation between language and music processing is particularly strong for rhythmic skills (Strait et al., 2011). More precisely, rhythm production skills turned out to be predictive for expressive grammatical abilities in school children (Gordon et al., 2015) and the ability to synchronize to a given beat predicted performance in pre-literacy skills such as phonological awareness (PA) and verbal memory in preschool children (Carr et al., 2014). In summary, prior evidence suggests that language abilities profit from musical abilities and demonstrate that a musical training has short- and long-term effects on important pre-literacy skills, such as PA (Degé and Schwarzer, 2011; Moreno et al., 2011). Theoretically, most of these studies have emphasized that musical training fosters children’s perceptual skills and enables them to process auditory material with higher precision (cf. OPERA-hypothesis; Patel, 2012). It has been argued that better auditory processing skills in turn lead to an enhanced processing of speech. Recent neurophysiological studies showed a higher proficiency of musicians compared to non-musicians in the pre-attentive encoding of low-level speech sounds (spectrally or temporally manipulated phonemes and vowels) as well as superior perceptual discrimination of subtle differences in pitch, duration and timbre of sine wave tones and vowels (e.g., Kühnis et al., 2013). Taken together, these findings suggest that differences in lower level speech perception between musicians and non-musicians may arise from a generic constitution of the auditory system rather than from speech specific representations. According to this view, transfer effects of musical training should be particularly strong for low-level auditory and phonological skills. In line with that, the meta-analysis by Gordon et al. (2015) suggests that transfer effects from a musical training on PA are small (*d* = 0.20) but relatively robust, even after controlling for hours of training and type of control intervention. In this context, previous studies point toward possible interfering variables such as musical expertise (cf. Gordon et al., 2015) that have to be taken into account when investigating music competencies and transfer effects. Other meaningful factors are represented by (non-verbal) intelligence (e.g., Schellenberg, 2005) as well as executive function (e.g., Degé et al., 2011) that seem to play a mediating role within the interrelation of language and music competencies.

In sum, previous studies have shown relationships between different competencies on various levels and emphasized the constitutional advantage of enhanced auditory processing skills derived from musical training. However, a comprehensive description of the interrelations between different language- and music-related competencies is still lacking and results remain fragmentary. This is particularly relevant because findings have important educational implications. At present, it is unclear which competencies exactly correlate and might promote the development of language skills across kindergarten and preschool.

### Acquisition of Language and Music Skills

There are important parallels between theories of the development of language and music (Chen-Hafteck, 1997; Brown, 2001; Kraus and Slater, 2015). In particular, in both domains a hierarchical model of step by step skill acquisition during childhood has been suggested. For the music domain, a chronological order of skill acquisition has been proposed (see e.g., Shuter-Dyson and Gabriel, 1981; Dowling, 1982): First, young children seem to be able to discriminate and react to different characteristics of sound (e.g., frequency; Bridger, 1961); next, from age five and upward children acquire the ability to discriminate between same and different melodies and rhythms as well as to repeat them; in a last step at the age of six and hereafter they seem to establish a harmonic sense (Krumhansl and Keil, 1982). Although infants showed very early the ability to recognize changes in melodic contour and range, the recognition of tonality and pitch seem to occur in later stages of infancy (Trehub et al., 1984). Thus, young children have rather global discrimination abilities and are able to differentiate between identical and non-identical tones. However, with increasing age and musical experience, they develop more differentiated perceptive skills in preschool (Sergeant and Boyle, 1980; Trehub et al., 1984). The same applies for the control of pitch during music production which seems to be one of the abilities in the course of musical development that are developed last (Trehub et al., 1984). Previous studies suggest a chronological order in the acquisition of song production, as well. At first, children are able to reproduce the words, second the rhythm, third the melodic contour before they are finally able to reproduce the intervals accurately (Davidson et al., 1981). More generally, previous research points toward a difference between the acquisition of perceptive and productive skills. As proposed by Dowling (1982) there might be different kinds of musical schemata, one referring to music perception, one to music production and/or one referring to both.

Similarly, theories of language development also assume that different skills are acquired in a chronological order (cf. Bloom, 1998; Jusczyk and Luce, 2002). For example, models of spoken language comprehension assume that processing is hierarchically organized, with reference to a greater abstraction of acoustic features of speech at higher processing levels (e.g., Davis and Johnsrude, 2003). Research points toward the ability to discriminate between different vowel contrasts and speech sound patterns (phonetic level) in early infancy and the subsequently establishing ability to discriminate between identical and non-identical words (morphemic level) (Trehub, 1993; Jusczyk and Aslin, 1995). Following that, passive vocabulary expands and children are supposed to be able to segment words from fluent speech until they are able to recognize the meaning of successive words in a sentence (syntactic level). Similar to music, previous research of language acquisition revealed an imbalance of perceptive and productive abilities in young children is that production seems to follow perception (e.g., Bates, 1995). Although evidence is inconclusive, it has been assumed that perceptive skills are necessary but not entirely sufficient for the production of speech (Bates and Goodman, 1999). Only few studies have addressed the question of similarities and differences in the production and perception of language and music so far. Existing evidence, however, indicates that there might be similar processes in terms of incremental planning in sequence production (Palmer and Pfordresher, 2003).

### Distinguishing Levels of Cognitive Processing in Language and Music

As the acquisition of both music- and language-related skills has been described as a sequence of hierarchically organized levels, it can be assumed that these levels might build upon each other. Indeed, there is evidence from neuropsychological studies in the domain of spoken language perception that language is processed on different cognitive levels (e.g., Besson and Schön, 2001) and that music processing on different levels is associated with distinct brain regions (Koelsch et al., 2005). In addition, there seems to be an overlap in structural processing between language and music. For example, the processing of music-related syntactic information [harmonic progression (HP)] showed activation in a cortical network that has been thought to be domain-specific for language processing (Koelsch et al., 2002). The syntactic processing of language and music stimuli even seems to compete for the same cognitive resources when being presented at the same time (Fedorenko et al., 2009). Moreover, a direct comparison of the production of linguistic and melodic phrases showed activations in similar functional brain areas indicating shared neural systems for phonological generativity (Brown et al., 2006). On the other hand, there is evidence leading toward activation of distinctive brain areas for the processing of other features, as e.g., semantics (Brown et al., 2006). In conclusion, it is to be assumed that there are specific overlaps between language and music as well as distinctive processing systems.

To summarize, previous studies indicate that it is reasonable to assume that both language and music skills can be systemized in different levels of cognitive processing which rely on units of various stimulus length. However, it is still unclear how exactly performances on different levels are interrelated and whether performance on lower levels is predictive for higher level processing.

### Rationale of the Study

The present study addresses some of the limitations of earlier studies by providing a systematic description of the interrelationships between language and music skills and by including a broad variety of language and music competencies representing different types of tasks (e.g., perceptive, productive). In addition, we aim to clarify whether it is appropriate to group language and music skills in distinct levels according to the size of units that are relevant for processing (e.g., phoneme, word, sentence, text). In particular, we suggest to group skills according to the following five different levels (see also **Table 1**): Level 1 is based on small perceptual units such as vowels and phonemes [phonemic discrimination (PD), word discrimination (WD)] and sounds and short tonal phrases [sound discrimination (SD), tonal discrimination (TD)]. Level 2 comprises skills that are relevant for the perception and production of syllables and words [phonological awareness (PA), prosodic repetition (PR)] and short rhythms and melodies [rhythmic repetition (RR), melodic repetition, (MR)]. Level 3 pertains to the processing of syntactic or harmonic sequences [syntactic integration (SI), harmonic progression (HP)]. Level 4 also taps into the processing of sequential units but particularly focuses on the recognition of emotions in spoken sentences or tonal phrases. Finally, Level 5 includes processes that represent high-level units such as stories or songs. We were particularly interested in the relationships between children’s performances on different levels both within and across domains. However, tasks can also be grouped into productive (Levels 2 and 5) and receptive tasks (Levels 1, 3, and 4).

**Table 1:** Accuracy values for the performance in language and music competencies of young children (5–7 years old) compared to young adults (20–30 years old) ordered by their associated levels of cognitive processing.

	Children	Adults	
			
	*M* (*SD*) accuracy	*M* (*SD*) accuracy	*t*
**Language**			
Level 1			
Phonemic discrimination (PD)	0.71 (0.12)	0.86 (0.08)	4.65^∗∗^
Word discrimination (WD)	0.95 (0.06)	0.99 (0.01)	4.56^∗∗^
Level 2			
Phonological awareness (PA)	0.93 (0.09)	0.99 (0.02)	3.22^∗∗^
Prosody repetition (PR)	0.92 (0.10)	0.98 (0.03)	2.48^∗∗^
Level 3			
Syntactic integration (SI)	0.78 (0.11)	0.98 (0.03)	7.56^∗∗^
Level 4			
Emotion recognition (ERs)	0.80 (0.22)	0.98 (0.04)	3.44^∗∗^
Level 5			
Narrative Comprehension (NC)	0.63 (0.17)	0.87 (0.06)	8.27^∗∗^
**Music**			
Level 1			
Sound discrimination (SD)	0.70 (0.20)	0.90 (0.09)	3.96^∗∗^
Tonal discrimination (TD)	0.75 (0.17)	0.95 (0.05)	4.81^∗∗^
Level 2			
Rhythm repetition (RR)	0.73 (0.27)	0.95 (0.12)	3.32^∗∗^
Melody repetition (MR)	0.45 (0.36)	0.67 (0.31)	2.08^∗^
Level 3			
Harmonic progression (HP)	0.63 (0.15)	0.82 (0.19)	4.21^∗∗^
Level 4			
Emotion recognition (ERt)	0.70 (0.24)	0.67 (0.12)	-0.57
Level 5			
Synchronization (SY)	0.53 (0.23)	0.73 (0.27)	3.32^∗∗^


*Final accuracy values consisted of the summarized score overall items divided by item number. Normalized values are available for the three PA subtests (BISC; Jansen et al., 1999) and the TD task (PMMA; Gordon, 1979). The children sample reached values comparable to 49.7, 52.0, and 61.9% of a norm sample for the three PA subtests “sound-to-word,” “sound association,” “syllable segmentation,” and a percentile rank of 91 for the TD task. Significant differences based on a two-sample *t*-test between the accuracy of children and adults are marked with ^∗∗^*p* < 0.01 and ^∗^*p* < 0.05 and reached large effect sizes from Cohen’s *d* = 0.65 (Melody repetition) to *d* = 1.88 (Narrative comprehension). Scale reliability for language competencies reached a Cronbach’s α = 0.79 and for music competencies α = 0.74.*

In line with previous findings, we hypothesized that language and music competencies in young children show relationships within each domain such that lower level performance predicts higher level performance (hypothesis 1). It is to be assumed that children in transition from kindergarten to school age already developed skills representing lower processing levels (e.g., phoneme/sound, syllable/rhythm) and are amidst their acquisition of skills representing higher processing levels (e.g., sentence/harmony, text/song). Hence, improved lower level processing skills should be predictive for a better performance in higher level processing skills. Secondly, we hypothesized significant correlations between both domains with regard to distinct processing levels (hypothesis 2). More precisely, we expected to find direct evidence for the theoretically derived associations between competencies representing the same level of cognitive processing (e.g., phonemic and SD, SI, and HP).

## Materials and Methods

### Sample and Procedure

Forty-four 5- to 7-year-old children (53.50% female; *M*_age_ = 6.51, *SD* = 0.77) participated in a computer-assisted study in Berlin, Germany. During the months of July and August 2015, each child was tested in three individual sessions á 40 min within a period of no longer than 1 week in between in their daycare facilities. Additionally, a control group consisting of 20 young adults (45% female; *M*_age_ = 26.14, *SD* = 2.82) worked on the same tasks individually in one 120 min session at the Max Planck Institute for Human Development in Berlin, Germany. The content and procedure of this study was approved by the ethics committee of the Max Planck Institute for Human Development in Berlin, Germany.

Participation in this study was voluntary and children were recruited via three different daycare facilities. Parents were informed by letter and signed a consent form for their child and filled out a questionnaire with demographic and home environmental information. Both parents gave information about their current occupation status and type and the ISEI index was coded from the parent with higher status, only. The parent’s socioeconomic background of our children sample was relatively high according to the International Socio-Economic Index of Occupational Status (ISEI) (*M* = 67.05, *SD* = 17.11). All participants indicated German as their first language and were born or living in Germany no later than from the age of three. Children were rewarded with a little gift after every testing session and young adults obtained an expense allowance of 15 EUR.

All computer-assisted tasks were presented with the experimental software Inquisit version 4.0.8.0 on a Dell laptop D 520 and via a Sennheiser PC 2 chat headset. Verbal answers in productive tasks have been recorded with the help of a Behringer Xenyx 203 USB mixing device and the corresponding software HarddiskOgg. Fifteen percent of the data from productive task types have been selected randomly and were blind coded by an additional independent rater. The inter-rater reliability varied from κ = 0.68 to κ = 0.93, *p*s < 0.01. Unless specified differently, during receptive discrimination tasks participants were asked to indicate whether presented stimulus pairs were same vs. different, correct vs. incorrect or happy vs. sad via two differently tagged keys on a keypad. In order to ensure that children understood the tasks, two to four practice trials were included.

### Measures

#### Control Variables

##### Non-verbal intelligence (IQ)

As an indicator for non-verbal intelligence (IQ) we used the matrices subtest from the German version of the Culture Fair Intelligence Test (CFT 1-R; Weiß and Osterland, 2013; adapted from Cattell and Cattell, 1973). The matrices subtest includes 15 items and the processing time is limited to 180 s in total.

##### Executive function (EF)

To assess executive function (EF) we used a go/no-go task indicating non-verbal inhibition as used by Moreno et al. (2011). Geometric shapes were presented randomly for 800 ms each on a computer screen while white colored shapes indicated the go-trials and purple colored shapes indicated the no-go trials. For a total of 60 trials separated by a blank screen interval of 500 ms, participants had to press a key on go trials and not on no-go trials.

##### Home literacy environment (HLE)

The parents of our children sample answered 12 items from the home literacy environment (HLE) Scale (cf. Niklas and Schneider, 2013) indicating literacy activities at home (e.g., “At what age in months did you begin to read to your child?”; “How many books for pleasure do you read per month?”).

##### Home musical environment (HOME)

As a measure of musical expertise we used the home musical environment (HOME) scale from Brand (1985) including 13 items answered by the parents (e.g., “We use music in our daily routine”; “My child plays a musical instrument”) on a 4-point rating scale from 0 = “never” to 3 = “frequently.”

#### Language Competencies

##### Phonemic discrimination (PD)

Based on the experiment from Groth et al. (2011) we compiled 24 stimuli with varying vowel length with the speech synthesis software Mbrola (Dutoit et al., 1996). Phonemes were presented in pairs of identical monosyllabic pseudowords or differing in length of the vowel, only (e.g., /fip-fi:p/, /pam-pa:m/). Participants were asked to indicate whether words were the same or different.

##### Word discrimination (WD)

The ability to discriminate between words was assessed with a task consisting of 32 pairs of similar but semantically differing monosyllabic words. Half of the stimuli were same (e.g., “Bar–Bar”; “Gast–Gast”) and the other half differed in their onset or offset (e.g., “Bar–Bart”; “Ast–Gast”).

##### Phonological awareness (PA)

Phonological awareness was assessed with three subtests consisting of 10 items each from a German screening inventory for the early detection of dyslexia (BISC; Jansen et al., 1999). In the “syllable segmentation” subtest participants were asked to repeat given words and indicate phonetic syllables by clapping simultaneously, in the “sound-to-word” subtest participants were asked to decide whether an isolated vowel is a constituent part of a subsequently specified word and during the subtest “sound association” the participants are asked to find the corresponding item to a given word on a pictorial map with four items.

##### Prosodic repetition (PR)

Based on the 18 stimuli from the subtest “phonological working memory of pseudo words” from the German test of speech development (SETK3-5; Grimm, 2001) we measured the participants’ ability to reproduce pseudo words with correct prosody. Items consisted of two to five syllables and we added an irregular intonation (emphasis on the second or third syllable which is rather untypical in the German language) for half of the items. Participants were asked to repeat each given word as precisely as possible.

##### Syntactic integration (SI)

The participant’s ability to identify grammatically correct and incorrect sentences was measured with 40 randomly presented items from the syntactic subtest of the German diagnostic of process-related reading and auding (ProDi-H; Richter et al., 2012).

##### Emotion recognition in spoken phrases (ERs)

To measure the participants’ ability to recognize emotions in spoken phrases we used eight stimuli from the Florida Affect Battery (Bowers et al., 1991) and also included their German translations spoken by one of the authors. Based on the results from Thompson et al. (2004) we included happy and sad sentences with neutral content (e.g., “The box is in the kitchen”). Consequently, the total amount of 16 sentences contained eight sentences with a happy and eight with a sad intonation and half were in English and the other half in German language.

##### Narrative Comprehension (NC)

With the narrative comprehension (NC) task we measured the participant’s competency to recognize and establish coherences in a story using pictures. Similar to Silva and Cain (2015) we used the following procedure: First, participants were presented with a picture book and were instructed to individually go through the book and explain to the experimenter what had happened in the story. After that, participants were asked to answer questions about the contents of the story (e.g., “Who are the characters in the story?”) or inferences about it (e.g., “What do you think the woman would be saying here?”). Answers were transcribed and coded with 0 = “no answer or inappropriate answer,” 1 = “includes an appropriate answer but it is not connected to other events/pages” and 2 = “an appropriate answer is identified and connected to other events/pages.”

#### Music Competencies

##### Sound discrimination (SD)

To measure the subtle perception of timbre differences in single tones we used 16 stimuli that have been generated and used by Grey (1977). The stimuli have been synthesized based upon a sound analysis of actual instrument tones (oboe, horn, clarinet, saxophone, trombone, and cello) and were equalized for pitch, loudness and duration (pitch E^b^ above middle-C, 311 Hz, duration between 280 and 400 ms).

##### Tonal discrimination (TD)

Participants’ ability to discriminate between same or different tonal sequences was measured with the tonal subtest from the Primary Measures of Music Audiation in young children from Gordon (PMMA; 1979) including 40 synthesized two- to four-tone tonal sequences presented with a piano sound.

##### Rhythm repetition (RR)

Rhythmic repetition was measured using a task from Sallat (2008). Similar to Jentschke et al. (2008) we used six rhythms consisting of three to six eighth and quarter notes. Stimuli were presented as a clapping sound in same tempo (80 bpm) and with increasing difficulty (length). Participants were asked to repeat the rhythm verbally by using the syllable “ta.” The accuracy of the responses was coded with 0 = “incorrect,” 0.5 = “partly correct,” and 1 = “correct” for each item and the ratio from total score and item number build the final rhythmic repetition accuracy value. The “partly correct” option was coded in case of a correct but incomplete repetition or when at least half of the rhythmic contour has been repeated correctly.

##### Melody repetition (MR)

The ability to repeat melodies was assessed with six items consisting of four to eight eighth and quarter notes similar to Sallat (2008). Half of the items represented rather familiar melodies from children’s songs and the other half were slightly modified by the authors and thus represented rather unfamiliar melodies. Stimuli were presented in terms of a programmed piano sound in the same tempo (80 bpm) and with increasing difficulty (length). Participants were instructed to repeat the given melody with the help of the syllable “la.” Coding of the accuracy in melodic repetition was equal to the rhythmic repetition with 0 = “incorrect,” 0.5 = “partly correct,” and 1 = “correct” for each item and the ratio from total score and item number build the final melodic repetition accuracy value. Again, in case of incomplete but correct repetitions or when at least half of the melodic contour has been repeated correctly the “partly correct” option was coded. Absolute pitch has been disregarded in behalf of accuracy in the relative melodic contour.

##### Harmonic progression (HP)

The competency to discriminate between regular and irregular HP was measured with ten items from Jentschke and Koelsch (2009). Items consisted of a sequence of five chords with either a regular tonic or irregular supertonic (Neapolitan chord) ending and participants were asked to decide whether the stimulus ending sounded correct or incorrect.

##### Emotion recognition in tonal sequences (ERt)

For the purpose of comparability between the recognition of emotions in language and music we included tonal sequences as produced and used by Thompson et al. (2004). On the basis of spoken sentences from the Florida Affect Battery (Bowers et al., 1991) Thompson et al. (2004) constructed tonal sequences mimicking the prosody of spoken sentences with regard to the modal pitch and duration (e.g., happy sequences were relatively quick and with a wide pitch range). In our study, we included eight tonal sequences analogous to the happy and sad sentences.

##### Synchronization (SY)

With a selection of three songs from Cohrdes et al. (2014) we measured the participant’s competency to tap along with a given music excerpt (30 s). Three songs (Pietro Lombardi – “Call my name”; J. S. Bach – “Suite Nr.3, Gavotte I”; Ursoaica – “Carnavalito”) were selected based on criteria of a high motion potential and high affiliation as rated by children in primary school age as well as varying tempo (slow, medium, high) and genre (pop, classic, world music). Participants were asked to tap along with the music in a way that seemed appropriate to them with a “rhythm key” on the keyboard that gave a clapping sound as auditory feedback. Coding based on the criteria of accuracy (which was based on the correspondence between the clapping and the meter of a given song) and was coded from 0 = “randomly hit,” 0.5 = “partly hit,” 1 = “consistently hit” and complexity (which was based on whether rhythmic extensions beyond the easy patterns were used, e.g., with variations in eighth and sixteenth notes or spread over multiple bars) as coded grading from 0 = “no pattern,” 0.5 = “easy pattern,” and 1 = “sophisticated pattern.”

## Results and Discussion

Results are organized in three different, but logically linked sets of analyses. In a first step, we compared the children’s and adults’ performances with each other in order to examine developmental differences (cf. section “Children’s and Adults’ Performance in Language and Music Tasks”). In the following analyses, we focused on the children group and briefly reported descriptive statistics for the control variables used in our study at the beginning of Section “Discrimination of Language and Music Competencies on Distinct Levels.” Furthermore, we explored in section “Discrimination of Language and Music Competencies on Distinct Levels” whether different measures can be grouped according to predefined processing levels and whether skills on lower levels are able to predict performance on higher levels. In section “Correlation of the Children’s Accuracy in Language and Music Competencies,” by contrast, we analyzed the correlations between language and music variables on the predefined five processing levels (section “Correlation of the Children’s Accuracy in Language and Music Competencies”). We included non-verbal IQ and EF as well as the HLE for language-related performances, respectively, the HOME for music-related performances as control variables in our analyses.

### Children’s and Adults’ Performance in Language and Music Tasks

To investigate how well children were able to perform the different tasks, we compared their response accuracy scores with that of adults. Descriptive statistics and *t*-tests are provided in **Table 1**: First, children showed a better than chance performance (>00.50) for all measures, indicating that they understood the instructions and were generally able to perform the tasks. Indeed, there were slight ceiling effects for some of the language-related measures (PA, WD, and PR) which indicate that these tasks were already too easy for our target group. Next, as expected, young adults generally showed significantly higher accuracy scores for both language and music skills with only one exception, the recognition of emotions in tonal sequences. The accuracy in the recognition of emotions in English and German spoken phrases did not differ significantly between participants, *t*(58) = -0.44, *p* = 0.66 indicating that both the English original stimuli as well as the German translations expressed the same emotional content that was recognized with an accuracy comparable to other studies (Thompson et al., 2004). The Cronbach’s alpha coefficient of internal consistency was α = 0.80 for English phrases and 0.79 for German phrases.

For both age groups, music-related tasks were more difficult than language-related tasks, *t*(58) = -8.29, *p* < 0.001, *d* = 1.60. For adults, the mean accuracy was *M* = 0.95 (*SD* = 0.02) for language-related tasks and *M* = 0.81 (*SD* = 0.12) for music-related tasks. For children, the mean accuracy was *M* = 0.82 (*SD* = 0.18) for language-related tasks and *M* = 0.64 (*SD* = 0.08) for music-related tasks. In addition, music-related productive tasks (in particular melodic repetition and synchronization) were more challenging for both children and adults than perceptive music tasks (cf. **Table 1**), *t*(58) = -3.43, *p* < 0.001, *d* = 0.49, indicating that the performance of productive music skills might afford specific training.

In sum, the results show that the difficulty of the selected tasks was appropriate for young children in transition from kindergarten to school age. However, they also showed sufficient variability to investigate developmental and inter-individual differences as children have not yet reached adult-like performance levels. Music-related tasks (and especially productive tasks) were generally more difficult than language-related tasks.

### Discrimination of Language and Music Competencies on Distinct Levels

In a next step, we focused on inter-individual differences in the children group. To investigate interrelationships between language and music skills which tap into the processing of units of different levels (e.g., vowel/sound, sentence/harmony; see above), we fit a series of hierarchical regression models for both domains, separately. We were particularly interested in whether the performance on lower levels was predictive for the performance on higher levels. To this end, we estimated a separate regression model for each outcome variable in each domain representing one of the five levels of processing. In each model non-verbal IQ, EF, the HLE, respectively, HOME were included as control variables and all component skills on lower levels. Results are summarized for each domain separately in **Table 2.**

**Table 2:** Results of multiple regression analyses predicting language and music performance on a higher level from lower level performance and in control of non-verbal intelligence (IQ), home musical environment (HOME), home literacy environment (HLE), and executive function (EF).

		Language			Music		
			
Level – Competencies	Model	*B*	*SE*	*t*	*B*	*SE*	*t*
1 – PD, WD/SD, TD	Intercept	0.70	0.19	**3.56^∗∗^**	0.30	0.33	0.91
	IQ	0.005	0.0023	1.86	0.01	0.005	1.92
	HLE/HOME	0.002	0.004	0.52	0.003	0.003	1.06
	EF	0.06	0.18	0.33	0.98	0.36	**2.66^∗^**
2 – PA, PR/RR, MR	Intercept	0.79	0.21	**3.79^∗∗^**	0.12	0.45	0.28
	IQ	0.009	0.002	**3.80^∗∗^**	0.03	0.007	**3.58^∗∗^**
	HLE/HOME	0.000	0.004	0.08	0.02	0.003	**4.47^∗∗^**
	EF	-0.15	0.16	-0.09	-0.81	0.54	-1.50
	Level 1	0.09	0.15	0.59	0.78	0.22	**3.48**^∗∗^
3 – SI/HP	Intercept	-0.41	0.36	-1.13	0.43	0.30	1.41
	IQ	0.002	0.004	0.48	-0.006	0.006	-1.07
	HLE/HOME	-0.003	0.006	-0.55	-0.01	0.003	**-3.37** ^∗∗^
	EF	0.08	0.24	0.32	0.34	0.38	0.91
	Level 1	0.58	0.14	**2.76^∗∗^**	-0.20	0.18	-1.11
	Level 2	0.71	0.16	**3.02^∗∗^**	0.64	0.12	**5.09^∗∗^**
4 – ERs/ERt	Intercept	1.15	0.85	1.34	0.15	0.60	0.25
	IQ	0.03	0.01	**2.94^∗∗^**	0.01	0.01	1.02
	HLE/HOME	-0.002	0.13	-0.13	0.002	0.007	0.22
	EF	-0.42	0.56	-0.76	-0.32	0.72	-0.43
	Level 1	0.31	0.57	0.55	0.81	0.35	**2.31^∗^**
	Level 2	-0.57	0.64	-0.88	0.01	0.32	0.04
	Level 3	0.19	0.39	0.49	0.18	0.36	0.50
5 – NC/SY	Intercept	-2.16	1.04	**-2.08^∗^**	0.14	0.87	0.17
	IQ	-0.01	0.01	-1.11	0.007	0.02	0.39
	HLE/HOME	0.01	0.01	1.17	-0.01	0.01	-0.89
	EF	0.36	0.25	1.45	0.32	1.06	0.30
	Level 1	0.01	0.44	0.03	0.40	0.56	0.71
	Level 2	0.95	0.48	**2.00^∗^**	0.85	0.41	**2.47^∗^**
	Level 3	0.63	0.29	**2.16^∗^**	-0.73	0.52	-1.39
	Level 4	0.15	0.13	1.15	-0.06	0.28	-0.19


*Parameter values given in the table are non-standardized regression coefficients, standard errors and *t*-values for each predictor and intercept. Significant results are marked with ^∗∗^ for *p* < 0.01 and with ^∗^ for *p* < 0.05 and highlighted in boldface. Language and music competencies abbreviations on Level 1: PD, phonemic discrimination; WD, word discrimination/SD, sound discrimination; TD, tonal discrimination; on Level 2: PA, phonological awareness; PR, prosody repetition/RR, rhythm repetition; MR, melody repetition; on Level 3: SI, syntactic integration/HP, harmonic progression; on Level 4: ERs, emotion recognition in spoken phrases/ERt, emotion recognition in tonal sequences; on Level 5: NC, narrative comprehension/SY, synchronization.*

#### Children Sample Characteristics: Control Variables

On average, non-verbal IQ-scores of our sample were moderate with a mean score of *M* = 6.84, *SD* = 4.09 for the matrices subtest which is equivalent to a *T*-value of 49 (cf. Weiß and Osterland, 2013). In the go-/no-go inhibition task participants showed a relatively high accuracy of *M* = 0.90, *SD* = 0.06, on average. Musical expertise furthered in the home environment of our sample was moderate with a mean score of *M* = 20.41, *SD* = 5.59 (theoretical maximum score = 36), as was the HLE with a mean score of *M* = 28.61, *SD* = 2.41 and comparable values to a same-aged German reference group (cf. Niklas and Schneider, 2013).

#### Regression Analyses

In line with hypothesis 1, for the language domain results showed significant effects between levels. In particular, both vowel/WD (Level 1) and repetition of words/syllables (Levels 2) turned out to make significant independent contributions to SI skills (Levels 3). In addition, NC (Level 5) was significantly predicted by word- (Level 2) and sentence-level (Level 3) variables. By contrast, and rather surprisingly, performance on the word/syllable level (Level 2) was not predicted by vowel/WD (Level 1). Similarly, recognition of emotions (Level 4) seems to be a skill that is largely independent of other skills in the language domain.

For the music domain, results also showed clear interrelationships between all levels of processing, as expected. First, melody/RR (Level 2) was predicted by sound/melody discrimination (Level 1), and both skills were significantly related to harmonic processing (Level 3). Similar to language, recognition of emotions (Level 4) was more independent of other musical skills as it was only significantly predicted by SD (Level 1). Finally, performance in the synchronization task (Level 5) was significantly predicted by melody/RR skills (Level 2).

In sum, we found substantial interrelationships between different component skills within both the language and music domain. In addition, results provide evidence for a hierarchical order of skills in which competencies on lower levels are predictive for the performance on higher levels, as hypothesized in hypothesis 1.

### Correlation of the Children’s Accuracy in Language and Music Competencies

In a final step, we investigated relationships between competencies in the language and the music domain. **Table 3** shows the partial correlations (controlling for non-verbal IQ, EF, HOME, and HLE) between the different domains which are summarized and ordered according to the level of processing they involve. Significant correlations after Bonferroni correction to *p* < 0.002 are printed in boldface.

**Table 3:** Partial correlations between language and music competencies while controlling for non-verbal IQ, HOME, HLE, and EF.

	Music competencies				
	
	Level 1 – SD, TD	Level 2 – RR, MR	Level 3 – HP	Level 4 – ERt	Level 5 – SY
**Language competencies**					
Level 1 – PD, WD	0.19	0.22	0.13	0.22	0.21
Level 2 – PA, PR	0.01	**0.78^∗∗∗^**	0.36	-0.11	0.18
Level 3 – SI	0.33	0.20	**0.44^∗∗∗^**	0.16	0.11
Level 4 – ERs	**0.61^∗∗∗^**	0.34	-0.11	**0.46^∗∗∗^**	0.26
Level 5 – NC	0.13	0.21	0.08	-0.08	0.34


*We used the Bonferroni correction due to multiple comparisons (*n* = 25) and adjusted the α-level of significance to *p* < 0.002. Significant correlations are marked with ^∗∗∗^ for *p* < 0.002 and highlighted in boldface. Language competencies on Level 1: PD, phonemic discrimination; WD, word discrimination; on Level 2: PA, phonological awareness; PR, prosodic repetition; on Level 3: SI, syntactic integration; on Level 4: ERs, emotion recognition in spoken phrases; on Level 5: NC, narrative comprehension. Music competencies on Level 1: SD, sound discrimination; TD, tonal discrimination; on Level 2: RR, rhythm repetition; MR, melody repetition; on Level 3: HP, harmonic progression; on Level 4: ERt, emotion recognition in tonal sequences; on Level 5: SY, synchronization.*

In line with our hypotheses 2, we found significant relationships between skills in both domains while controlling for children’s non-verbal IQ, HOME, HLE, and EF. Since tasks in both domains are ordered hierarchically with ascending processing units, correlations on the diagonal represent the interrelationships between domains for tasks on same hierarchical levels. Notably, these correlations were generally higher than correlations between different levels. This indicates that performances on different levels do indeed share common variance across domains. Productive language competencies as the repetition of words and syllables on Level 2 significantly correlated with productive music competencies such as the repetition of melody and rhythm, as expected. Furthermore, correlations on the syntax level (Level 3) turned out to be significant between domains, too. Next, the competency to recognize emotions in spoken phrases (Level 4) correlated significantly with the competency to recognize emotions in tonal sequences and additionally with the competency to discriminate between low-level units as sound and tonal sequences (Level 1). Low-level competencies such as the discrimination of phonemes and sounds (Level 1) on the other hand did not correlate between domains indicating differences in the processing of language and music-related sounds but similarities between low-level music-related sounds and language-related emotional sounds (prosody).

In sum, our results indicate that there are substantial interrelationships between music and language skills in young children even after controlling for non-verbal IQ, HOME, HLE, and EF. These associations are stronger between tasks reflecting similar cognitive processes, as predicted in hypothesis 2.

## General Discussion

In the present study, we investigated the performance of young children in transition from kindergarten to school age in a comprehensive selection of language and music skills. At first, we compared the accuracy of our children sample to a young adult control group. Next, we grouped competencies of both domains according to distinct levels of cognitive processing relying on units of various lengths and conducted multiple regression analyses to test the predictability of performance on lower levels on the performance on higher levels within domains. In order to investigate the interrelationship between language and music competencies we next conducted partial correlation analyses between the predefined levels in control for non-verbal IQ, EF, HOME, and HLE.

In summary, selected competencies in this study turned out to be representative for both language and music and ensured a sufficient range of difficulty in both domains. With regard to developmental differences, young adults generally performed better than our target sample of young children but children already showed a better than chance accuracy and even comparable high accuracy values in the performance of a few language competencies (PA, WD, and PR). With regard to the competency of ERt we found no significant differences between young children and young adults pointing toward the question whether this competency improves with age, at all. The accuracy of our adult sample is comparable to results from Thompson et al. (2004) indicating that this task might be relatively specific and not appropriate to investigate age- and experience-related changes in the competency to recognize emotions within the music domain. Overall, however, results show that the difficulty of selected tasks was appropriate for young children and showed sufficient variability to investigate inter-individual differences. For future studies, we recommend a few improvements by including song excerpts instead of synthesized tonal sequences for the recognition of emotions in music as well as increasing the difficulty of the phonological discrimination and awareness tasks.

Generally, results indicate that language and music skills are significantly interrelated and furthermore that when comparing diverse competencies it is helpful to sort them systematically according to the level of cognitive processing they involve. Multiple regression analyses showed that specifically competencies on the syntactic level can be predicted by performance on lower levels in both language and music, as predicted in hypothesis 1. This finding suggests that the competency to integrate smaller units into a higher level syntactic system build upon the abilities to discriminate as well as reproduce smaller units as phonemes/sounds, words/tonal phrases, syllables/rhythmic phrases. Consequently, our results indicate similarities in the syntactic processing between both domains as suggested by the SI model from Patel (2003). Likewise, higher level processing in language (NC) seems to build upon abilities on the syntax level (Level 3) as well as on the words and syllables production level (Level 2). The synchronization task representing the music-related counterpart turned out to build upon productive skills as melody and RR (Level 2), too, but was independent of the performance on the syntactic level including HP. Generally, this indicates that competencies on higher levels might build upon different competencies on lower levels. This finding is in line with implications from neurophysiological studies indicating overlaps between specific language and music processes as well as rather independent or additional activation of brain areas representing higher levels of processing (e.g., Brown et al., 2006). Moreover, there seems to be a difference between productive and receptive task and their relevance for higher processing levels. This is particularly important because previous research did not systematically differentiate between these two types of tasks. Further research is needed in order to investigate potential differences between receptive and productive tasks in a developmental context.

Most importantly, we could show that there were substantial interrelationships between music and language-related skills in young children even after controlling for non-verbal IQ, HOME, HLE, and EF. Moreover, skills that tap into similar processes and involve comparable sized units in both domains were generally closer related than skills that involved different processes. This is in line with previous research that has compared similar competencies, as e.g., rhythm and PA (Moritz et al., 2013) represented on Level 2 in our study. With the results of our study we were able to show that it also holds for skills involving higher structural processing such as syntactic or contextual integration. Previous findings indicating a relationship between competencies on different levels as, e.g., to synchronize with a given beat and PA or other pre-literacy skills (e.g., Carr et al., 2014) could not be replicated with our study. This discrepancy might be related to the fact that in the present study, children were instructed to synchronize with a more complex musical structure (song) and not with a beat, only. In sum, one can conclude that competencies on the same level show higher correlations than compared to correlations between varying levels of cognitive processing.

However, perceptual tasks involving the processing of single phonemes/sounds and short words/tonal phrases (Level 1) were not related between domains indicating different processing or non-related perceptual skills. On the contrary, we found a significant correlation between lower level auditory processing skills (Level 1) and the recognition of supra-segmental elements in spoken sentences with varying emotional content (Level 4). This finding can be interpreted within the framework of interrelatedness between lower and higher levels of language processing (e.g., Bates and Goodman, 1999). In a review, Besson et al. (2011) argue that an increased ability at lower processing levels might affect the processing on higher levels. Following that, they conclude that an improved auditory processing based on musical training might also influence a higher level of speech processing. Until now, it remains unclear what exactly the music competencies are that have an impact on low- or high-level language competencies. Hence, it is an interesting perspective for future research to specify transfer effects from music on language on distinct levels (Besson et al., 2011). In addition, this finding seems to be in line with the OPERA hypothesis (Patel, 2012) which assumes that subtle auditory discrimination skills in the music domain have a positive effect on general discrimination skills which in turn promote language perception. Based on our results, this positive transfer effect might not pertain to the lower level phonological processing competencies as suggested by Patel (2012) but to higher processing levels. It would thus be helpful if future research would distinguish more clearly between different types of low-level perceptual processing and cross-domain effects longitudinally in order to investigate the cognitive locus and transfer of these effects.

## Conclusion

This study provides further evidence for interrelationships and differences between language and music skills in young children. In contrast to previous studies which typically used only few tasks and focused on very specific skills, we investigated potential correspondences between domains rather comprehensively by including tasks tapping into different levels of processing. Results show substantial correlations between language and music-related skills especially if similar processes are involved. This is an interesting starting point for future research in the field of language and music skill acquisition considering that the present study does not allow us to draw strong conclusions about underlying causal relationships that drive these associations. Future studies using longitudinal and experimental designs would be needed in order to explain the causal dynamics of the observed effects.

## Author Contributions

All authors substantially contributed to the present paper. The study was designed and planned by CC, LG, and SS. CC and LG performed the data collection, CC and SS analyzed the data. All authors discussed the results and implications. CC drafted the manuscript and LG and SS critically revised it. All authors approved the final version to be submitted and agree to be jointly accountable for all aspects of the work.

## Conflict of Interest Statement

The authors declare that the research was conducted in the absence of any commercial or financial relationships that could be construed as a potential conflict of interest.
